# Farnesyl pyrophosphate compartmentalization in the green microalga *Chlamydomonas reinhardtii* during heterologous (*E*)-α-bisabolene production

**DOI:** 10.1186/s12934-022-01910-5

**Published:** 2022-09-14

**Authors:** Julian Wichmann, Annibel Eggert, Liam D. H. Elbourne, Ian T. Paulsen, Kyle J. Lauersen, Olaf Kruse

**Affiliations:** 1 Faculty of Biology, Center for Biotechnology (CeBiTec), Bielefeld University, Universitätsstrasse 27, 33615 Bielefeld, Germany; 2 Department of Molecular Sciences, Macquarie University, Sydney, NSW Australia

**Keywords:** Microalgae, *Chlamydomonas reinhardtii*, Terpenoids, Isoprenoids, Bisabolene

## Abstract

**Background:**

Eukaryotic algae have recently emerged as hosts for metabolic engineering efforts to generate heterologous isoprenoids. Isoprenoid metabolic architectures, flux, subcellular localization, and transport dynamics have not yet been fully elucidated in algal hosts.

**Results:**

In this study, we investigated the accessibility of different isoprenoid precursor pools for C_15_ sesquiterpenoid generation in the cytoplasm and chloroplast of *Chlamydomonas reinhardtii* using the *Abies grandis* bisabolene synthase (*Ag*BS) as a reporter. The abundance of the C_15_ sesquiterpene precursor farnesyl pyrophosphate (FPP) was not increased in the cytosol by co-expression and fusion of *Ag*BS with different FPP synthases (FPPSs), indicating limited C_5_ precursor availability in the cytoplasm. However, FPP was shown to be available in the plastid stroma, where bisabolene titers could be improved several-fold by FPPSs. Sesquiterpene production was greatest when *Ag*BS-FPPS fusions were directed to the plastid and could further be improved by increasing the gene dosage. During scale-up cultivation with different carbon sources and light regimes, specific sesquiterpene productivities from the plastid were highest with CO_2_ as the only carbon source and light:dark illumination cycles. Potential prenyl unit transporters are proposed based on bioinformatic analyses, which may be in part responsible for our observations.

**Conclusions:**

Our findings indicate that the algal chloroplast can be harnessed in addition to the cytosol to exploit the full potential of algae as green cell factories for non-native sesquiterpenoid generation. Identification of a prenyl transporter may be leveraged for further extending this capacity.

**Supplementary Information:**

The online version contains supplementary material available at 10.1186/s12934-022-01910-5.

## Background

**Fig. 1:**
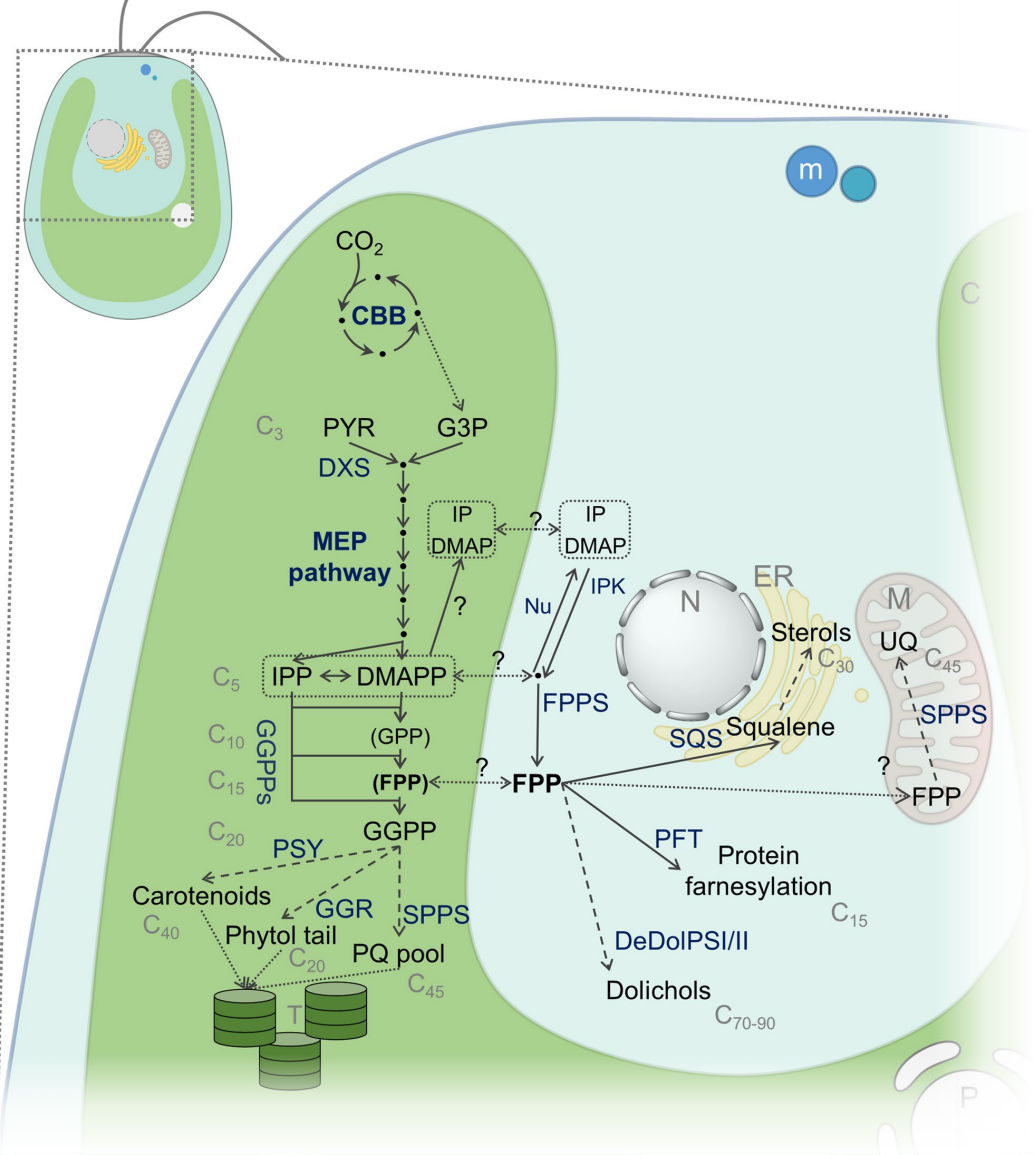
Isoprenoid pathways at the intersection of green algal compartments. Indicated are major intermediates (in black), enzymes (dark blue), reactions or reaction series (solid and dashed arrows, respectively), and transport (dotted arrows). CBB: Calvin Benson Bassham cycle; DXS: 1-deoxy-d-xylulose 5-phosphate synthase; PSY: phytoene synthase; GGR: geranyl geranyl reductase; SPPS: solanesyl pyrophosphate synthase; PQ: plastoquinone; Nu: Nudix superfamily; IPP: hydrolase; IPK: isopentenyl phosphate kinase; PFT: protein farnesyl transferase; SQS: squalene synthase; DeDolPS: dehydrodolechyl pyrophosphate synthase; UQ: ubiquinone; M: mitochondrion; LD: lipid droplet; N: nucleus; ER: endoplasmic reticulum; m: (peroxisomal) microbody; C: chloroplast; T: thylakoid; ? indicates unknown enzymes and transport mechanism(s). Parts of the figure have been created with BioRender.com

Eukaryotic algae and plants have evolved metabolic architectures enabling a photosynthetic lifestyle. Isoprenoids, also referred to as terpenoids, represent essential compounds involved in photosynthesis, including light harvesting and photoprotection (carotenoids, phytol-associated chlorophylls), charge separation (phytol-associated chlorophylls), as well as electron transfer (plastoquinone) (Fig. 1). In addition to the chloroplast, isoprenoids are intimately part of metabolism in the endoplasmic reticulum (sterols, dolichols) and mitochondria (ubiquinone), but also fulfill numerous specialized extracellular tasks such as defense, attraction, and signaling for plants [1]. Although isoprenoids are chemically highly diverse, their biosynthesis begins with modular condensation reactions of the C_5_ building blocks isopentenyl pyrophosphate (IPP) and dimethylallyl pyrophosphate (DMAPP) into higher chain length precursors which are derivatized into the different terpenoid branches, e.g. C_15_ sesquiterpenes, C_20_ diterpenes, C_30_ triterpenes, and C_40_ tetraterpenes. In plants, including algae, prenyl transferases catalyze these elongations according to need in various cellular compartments (Fig. 1) as predicted by genome sequencing and annotation [2].

The chemical complexity of isoprenoids is mediated by enzymes known as terpene synthases and subsequent chemical decoration is achieved by cytochrome P450s [3]. Many specialty terpenoid chemicals have found high-value applications for humans, such as the anti-cancer drug paclitaxel, or anti-malarial artemisinin. Specialty isoprenoid chemicals are often found in low abundances in their natural context, are difficult to synthesize by traditional chemistry, their precursors are found in all organisms, and may have numerous potential applications. These properties have led to the desire to produce specialty isoprenoids through metabolic engineering of microbial hosts that are easier to cultivate at scale. Engineering fermentative heterotrophs for isoprenoid production has developed into a mature technology, relying on high growth rates of these organisms on sugar or other organic carbon substrates to very high cell densities. Unconventional host organisms like eukaryotic green algae can offer several advantages to their fermentative counterparts [4] including a high basal turnover of precursors, consumption of inexpensive waste CO_2_ as a carbon substrate, and the high similarity of their metabolic structure and intracellular environments to plants [5]. In microalgae, metabolic precursors of cytosolic C_15_ farnesyl pyrophosphate and plastidic C_5_ as well as C_20_ geranylgeranyl pyrophosphate have been demonstrated to be freely available for conversion to heterologous terpenoid products by expression and localization of heterologous terpene synthases to respective cellular compartments [6–8].

Although green microalgae such as *Chlamydomonas reinhardtii* are closely related to land plants, their isoprenoid metabolism differs significantly: land plants have both the (plastidic) MEP and (cytosolic/peroxisomal) MVA pathway to synthesize IPP and DMAPP, green microalgae meet their prenyl unit supply solely using the MEP pathway [2]. This unique feature implies export of prenyl compounds from the chloroplast by a yet to be identified transport mechanism. It is still not understood which molecular species, C_5_ pyrophosphate, C_5_ monophosphate [9], and/or higher chain length (e.g. C_10_ / C_15_) prenyl moieties, are involved in this process.

The development of intron-mediated enhancement strategies for transgene expression [10, 11] has enabled *C. reinhardtii* to become a photosynthetic host for the overproduction of isoprenoid compounds from its different cellular compartments [6–8, 12]. Although it has become evident that the plastidic MEP pathway has a remarkable flux capacity towards C_20_ GGPP precursors for the production of diterpenoid products [6] and the FPP pool in the cytosol is tunable [8], only sparse knowledge is available regarding prenyl precursor presence and accessibility in different subcellular compartments as well as their dynamics under different carbon modes. In particular, sesquiterpene production capacity from the chloroplast has not been investigated but may be higher here than in the cytosol due to the prevalence of IPP and DMAPP in this compartment, specifically under phototrophic conditions [6]. The dynamics of FPP in the green algal cytosol have yet to be elucidated in detail, other than the ability to produce heterologous sesquiterpenoids by sesquiterpene synthases localized in this space [7, 8]. The FFPS of *C. reinhardtii* was found to have a peculiar localization as a halo structure around the nucleus although heterologous sesquiterpene was produced by a synthase freely floating around the cytoplasm [7].

The aim of this work was to enhance the efficiency of *C. reinhardtii* as a green cell factory for isoprenoid production by attempting to tap plastidic isoprenoid precursor pools for heterologous sesquiterpenoid synthesis and understand the dynamics of metabolism between the cytoplasm and chloroplast stroma. The *Abies grandis* bisabolene synthase (*Ag*BS) [13] was targeted to different cellular localizations and served as a metabolite pool reporter in conjunction with several fusion partners. Plastidic production of (*E*)-α-bisabolene was established and enhanced via different engineering strategies, and production dynamics during scale-up cultivation with different carbon sources and light regimes were investigated in a robust production strain. Bioinformatic methods were also employed to propose potential prenyl unit transporters, which may be responsible for our observations.

## Results

### FPP is available for sesquiterpenoid generation in the chloroplast

**Fig. 2:**
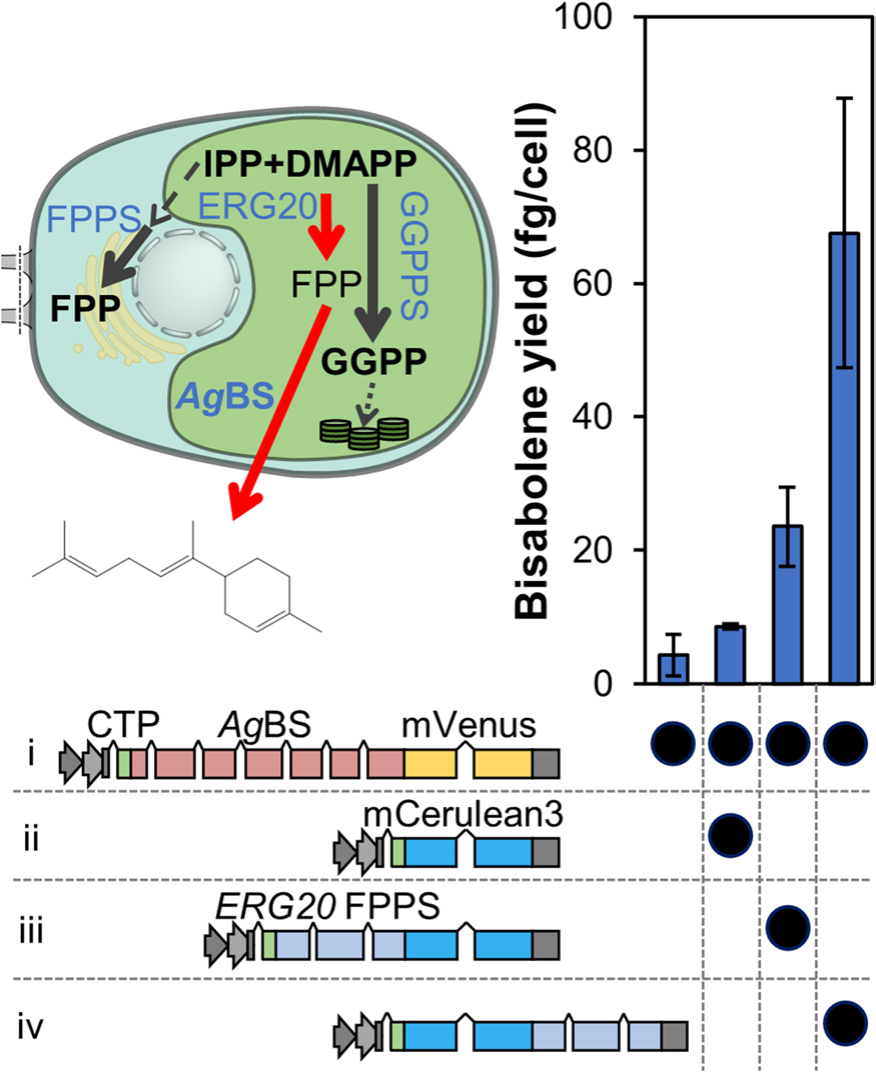
Native plastidic FPP can yield heterologous sesquiterpene synthesis by *Ag*BS-mVenus accumulation in the chloroplast. FPP pools and heterologous sesquiterpene titer can be increased by co-expression of *Sc*ERG20 in different orientations with the mCerulean3 reporter. Chloroplast targeting was achieved with the PsaD chloroplast targeting peptide (CTP). Bisabolene yields (fg cell^−1^) are depicted for the different combinations of constructs. Error bars represent 95% confidence intervals

**Fig. 3:**
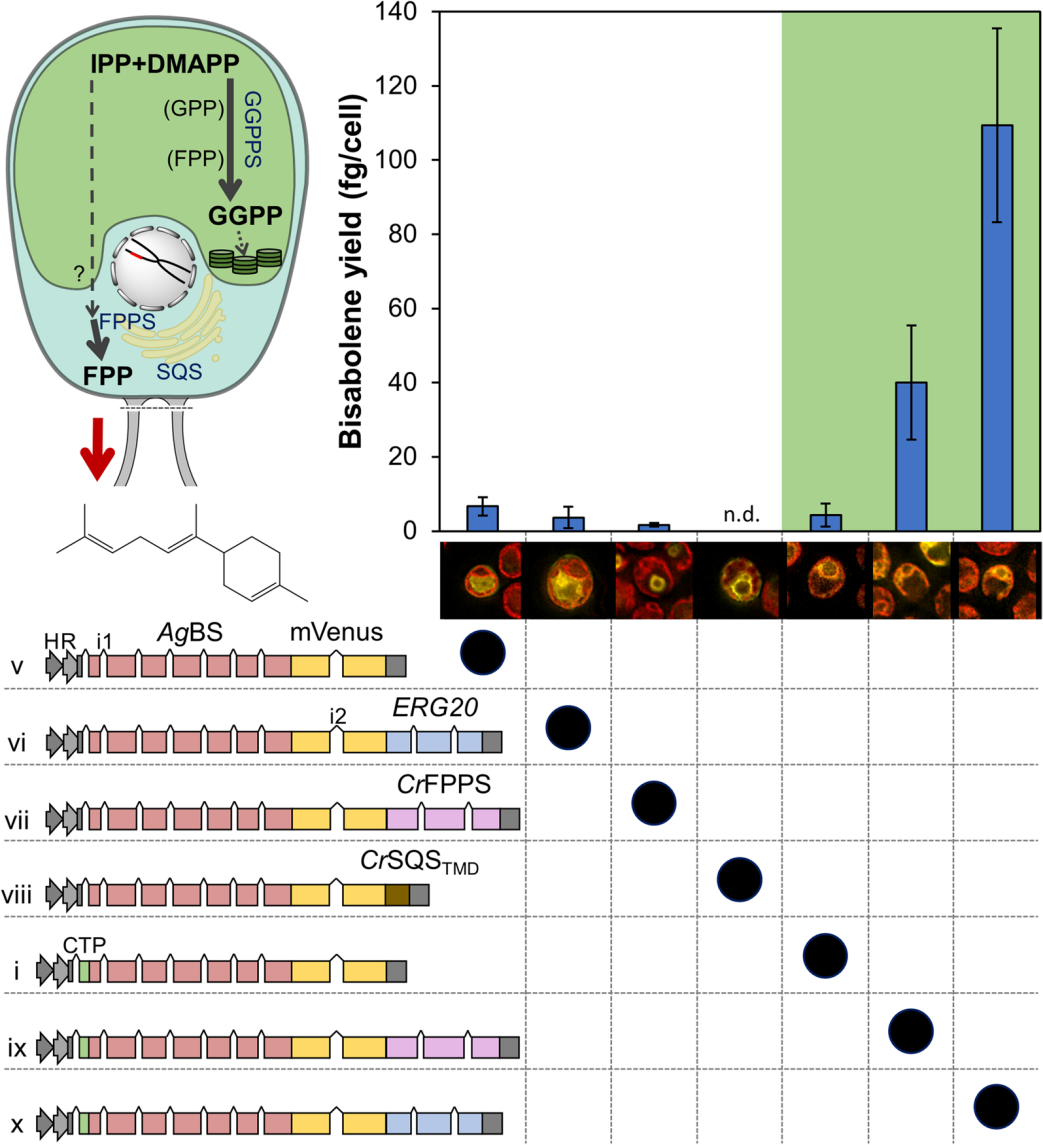
Differential subcellular targeting and its effect on bisabolene yields (fg cell^−1^). *Ag*BS-mVenus proteins were expressed as fusion to various enzyme partners and SQS transmembrane domain (TMD). Fusion protein localizations were confirmed by fluorescence microscopy. H Hsp70A promoter. R Rbcs2 promoter. i1 Rbcs2 intron 1. i2 Rbcs2 intron 2. Error bars represent 95% confidence intervals

To verify the presence of FPP in the stroma of the chloroplast, an *Ag*BS-mVenus fusion protein was targeted to this compartment using construct i (Fig. 2). Single-cell fluorescence microscopy (Fig. 3) and immunodetection (Additional file 11: Fig. S2) confirmed full-length expression of this protein to the plastid. Transformants produced minor yields (4.31 ± 3.13 fg cell^−1^) of (*E*)-α-bisabolene during two-phase cultivation with a dodecane overlay (Fig. 2). IPP and DMAPP precursors were previously shown to be abundantly available in the plastid for conversion to C_20_ GGPP and diterpenoid products [6], implying that these precursors could also be available for FPP generation. To use this potential, a highly active *S. cerevisiae* FPP synthase (*Sc*ERG20) was co-overexpressed as N- and C-terminal fusion to the mCerulean3 reporter. Compared to reporter-alone control transformants, cell lines with Western blot verified *Sc*ERG20 expression (Additional file 11: Fig. S2) exhibited elevated (*E*)-α-bisabolene yields (Fig. 2). The yield improvement was highest (~ eightfold higher, 67.52 ± 20.24 fg cell^−1^) when *Sc*ERG20 was fused to the C-terminus of mCerulean3 similar to its *Sc*ERG20(F96C) GGPPS variant [6]).

### IPP and DMAPP are abundant prenyl precursors only in the chloroplast

Enzyme fusions have been used as a valuable tool to harness substrate proximity/channeling effects for improved flux to terpenoid products [6, 14]. In this work, enzyme fusions of N-terminal *Ag*BS, an mVenus reporter, and a C-terminal fusion partner were either targeted to the cytosol or to the chloroplast in order to elucidate localization and precursor channeling factors with bisabolene yields as a reporter signal for metabolic pools (Fig. 3). A similar strategy including a *Coleus forskohlii* terpene synthase, a reporter gene, and the *Sc*ERG20(F96C) GGPPS as fusion partners targeted to the algal chloroplast previously resulted in increased diterpenoid production [6]. Fusion partners to the *Ag*BS investigated here included two FPPSs: *Sc*ERG20 and the native *C. reinhardtii* FPPS (*Cr*FPPS), as well as the transmembrane domain of the native *C. reinhardtii* squalene synthase (*Cr*SQS_TMD_, [15]). Targeting was directed to either the cytosol and the chloroplast mediated by the PsaD chloroplast transit peptide, with the *Ag*BS-mVenus as references in both cytoplasm and chloroplast. Subcellular localizations of the fusion proteins observed in single-cell fluorescence microscopy deviated from the reference constructs only for *Cr*FPPS and *Cr*SQS_TMD_ fusion partners when targeted to the cytosol, but confirmed previously detected localizations including a nuclear halo for *Cr*FPPS [7] and ER structures associated to the *Cr*SQS_TMD_ [15], respectively. Cell lines expressing fusion proteins targeted to the cytosol (vectors v-viii) produced only small amounts of bisabolene (< 10 fg cell^−1^) or none in the case of fusion partner *Cr*SQS_TMD_. Attempts to increase production from the cytosol by co-expressing the native *Cr*FPPS with *Ag*BS-mVenus also resulted in no improvements in titers (Additional file 11: Fig. S1). By contrast, fusion with either *Cr*FPPS or *Sc*ERG20 targeted to the chloroplast resulted in ninefold and 25-fold higher yields compared to the reference protein (*Ag*BS-mVenus) in this compartment, respectively (Fig. 3, vectors i,ix,x).

### Enzyme titer limits productivity from the chloroplast

**Fig. 4:**
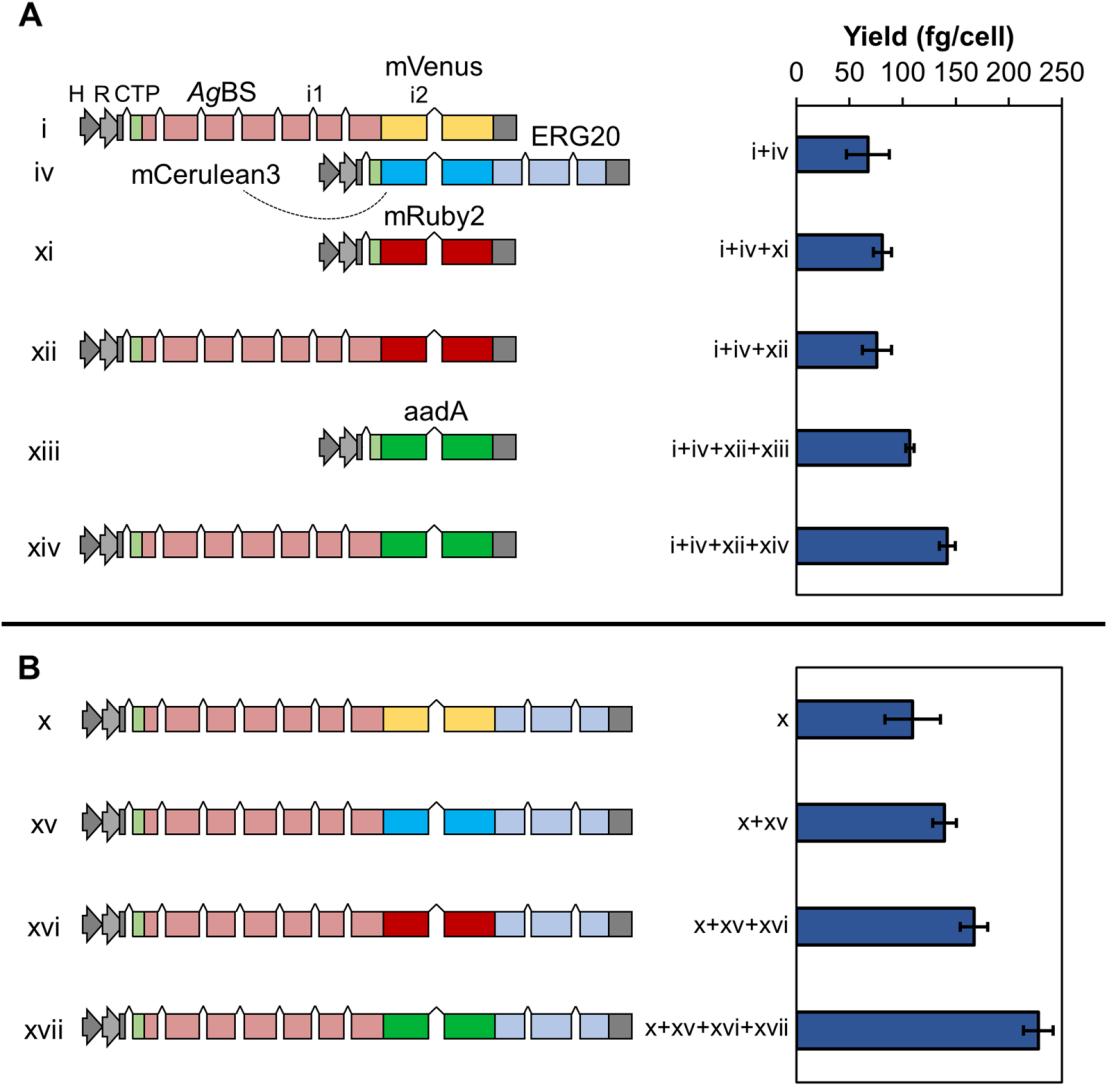
Enzyme accumulation by sequential transformations to enhance flux towards bisabolene in the chloroplast. **A** Co-expression of *Ag*BS and *Sc*ERG20 from separate constructs (i + iv), and serial transformation with further *Ag*BS copies or respective empty vector controls (left). Bisabolene yields (fg cell^−1^) are shown on the right. **B** Enhancement of fusion construct accumulation (*Ag*BS-reporter-*Sc*ERG20) using serial transformation of four separate constructs and correspondent product yields. Abbreviations as in Fig. 3. Error bars represent 95% confidence intervals

Low enzyme titers of catalytically inefficient terpene synthases often represent bottlenecks in metabolic engineering efforts, especially in algae [6, 8, 16]. To obtain more robust expression levels and accumulation of the best performing enzyme combinations, we conducted two transformation series with different reporter and selection markers fused to *Ag*BS/*Sc*ERG20 (Fig. 4). A co-expression strategy using mCerulean3-*Sc*ERG20 and serial loading with *Ag*BS-reporter fusions (Fig. 4A), as well as serial transformations with *Ag*BS-reporter-*Sc*ERG20 direct fusion constructs (Fig. 4B) were performed. Both strategies resulted in increased *Ag*BS protein accumulation (Additional file 11: Fig. S4) and similar moderate yield increases in bisabolene production (~ 2.1-fold) (Fig. 4A and B). The highest absolute values of bisabolene from a single transformant line (245 fg cell^−1^) were obtained with *Ag*BS-reporter-*Sc*ERG20 fusions (Fig. 4B).

### Influence of light regime and carbon source on plastid bisabolene titers.

**Fig. 5:**
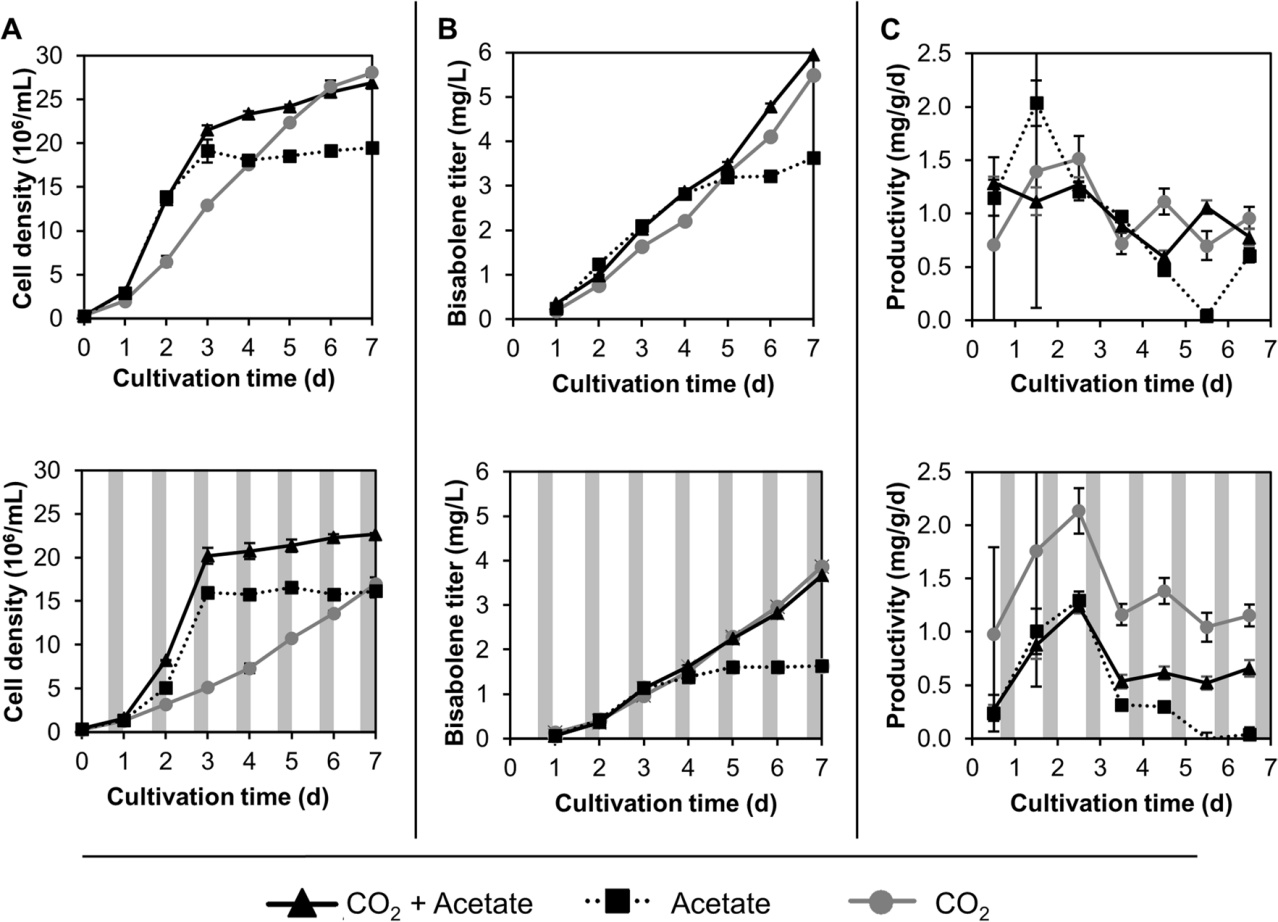
Growth and production dynamics of a robust performing strain expressing constructs x + xv + xvi. Cells were cultivated under different light and carbon conditions with light intensities of 100 μmol photons m^−2^ s^−1^ at 24 °C with a 5% dodecane overlay. Cell densities, bisabolene titers, as well as productivities were recorded daily. Grey columns indicate dark phases of 8 h. Error bars represent 95% confidence intervals

Production dynamics of a strain reaching robust bisabolene productivities (expressing constructs x + xv + xvi, Fig. 4B) were further analyzed in scale-up cultivations with different carbon regimes (3% CO_2_ + acetate, air + acetate, and 3% CO_2_). All cultivations were performed in constant light as well as in 16:8 h light:dark cycles with daily records of cell dry weight, cell densities, and bisabolene concentrations (Fig. 5). Growth proceeded with an exponential increase and a stationary phase in acetate-alone cultures, a linear increase in photoautotrophic cultures, and a combination of both in mixotrophic cultures (Fig. 5A). Light–dark cycles retarded these trends as previously observed [8]. Bisabolene titers followed similar patterns, although accumulating consistently in dodecane overlays. Bisabolene production in acetate-alone cultures nearly ceased ~ 1 day after reaching stationary phase in both conditions (Fig. 5B). Volumetric productivities were higher for constantly illuminated cultures than for those grown in light–dark regimes (Fig. 5B). While constantly illuminated cultures exhibited comparable productivities (mg/g/d), purely photoautotrophic cultures outperformed mixotrophy when normalized to the cell dry mass in day-night regimes (Fig. 5C).

### Identification of putative chloroplast prenyl exporters

Our results indicated that FPP to bisabolene yields could not be improved by the overexpression of native or heterologous FPPSs in the cytoplasm of *C. reinhardtii*. However, these enzymes mediated marked improvements in sesquiterpene yields in the chloroplast (Fig. 3). FPPSs use C_5_ IPP and DMAPP to make C_15_ FPP, and the abundance of C_5_ substrate in the chloroplast is expected as this is its site of synthesis. Our findings open a key question in isoprenoid metabolism of the alga; how does FPP find its way to the cytoplasm where production of sesquiterpenes has been shown [7, 8]? To identify transporter candidates capable of exporting prenyl units from the chloroplast, two strategies were applied. First, putative transporters identified by TransAAP were narrowed down to candidates localizing to the plastid membrane using DeepLoc and further refined to such transporters without known substrates using Blast/TransAAP. This left one protein sequence, PNW83272.1 (UniProt A0A2K3DRV6, Phytozome Cre05.g232751), which TransAAP annotated as an MFS transporter with substrate designation “multidrug efflux”, a generic description that may suggest the substrate is an amphipathic molecule like a pyrophosphate precursor. Putative transporters identified by TransAAP were also screened for the characteristic domain of prenyl transferases, Pfam UbiA HMM PF01040, however, both identified candidates have conserved domains with annotated functions, tocopherol phytyltransferase (NCBI) / homogentisate solanesyltransferase (Phytozome) for Cre06.g283750, and chlorophyll synthetase for Cre06.g294750.

## Discussion

### C_15_ FPP is present in the chloroplast

Green algae such as *C. reinhardtii* are eukaryotic photosynthetic organisms and must turn over all five prenyl diphosphate precursors (IPP, DMAPP, GPP, FPP, GGPP) needed for the synthesis of the most prominent isoprenoid classes. It is largely unknown in which abundance each product could be found in the algal cell depending on the cellular compartment and their metabolic role, and how these molecular species are transported across lipid bilayers [2]. The comparison of specific terpene synthase yields does not offer much insight here, as each synthase enzyme has its own rate of reaction, promiscuity of products, and different terpenoid products can exhibit toxicity. Metabolic architectures in similar systems such as land plants, and genomic as well as biochemical evidence for *C. reinhardtii* [2] suggest that the C_15_ FPP precursor pool would be available in the cytosol/ER of the algal cell as a precursor for sterol and dolichol metabolism as well as farnesylation of proteins, whereas C_20_ GGPP acts as a precursor for the synthesis of pigments and prenyl plastoquinones in the chloroplast (Fig. 1).

By targeting respective synthases to specific subcellular compartments of the algal cell, the generation of specific heterologous terpenoid products provided a facile confirmation of these metabolite localizations [6–8]. It was previously unknown if, and to which extent, FPP would be freely available in the chloroplast. In one previous investigation [17], it was attempted with no success to use FPP as a substrate in the chloroplast of *C. reinhardtii*. In our previous work [7], a patchoulol synthase was targeted to the algal chloroplast and mitochondria without generating the sesquiterpenoid patchoulol. However, this was not systematically investigated and yields of single enzyme expression may have been too low to detect the product. In our work here, we find strong evidence that this C_15_ hydrocarbon precursor is indeed present in the chloroplast (Figs. 2 and 3). FPP-derived C_15_ bisabolene was detected when targeting the respective terpene synthase (*Ag*BS) to the chloroplast, and titers were similar to strains with cytosolic targeting of this enzyme (Fig. 3, compare constructs v and i). It could be argued that the observed bisabolene production is from a small fraction of *Ag*BS remaining in the cytosol during import to the chloroplast. However, significantly lower sesquiterpenoid yields would be expected and no residual fluorescence signals could be detected in this compartment, implying this is likely not the case (Fig. 3). Similar results were obtained with higher plants: in moss, targeting of a santalene synthase and a patchoulol synthase to the chloroplast also resulted in the expected sesquiterpenoid products [18]. Although farnesyl pyrophosphate is likely present in the chloroplast, its function in this compartment is not clear. It may be the precursor to solanesyl diphosphate, used in the prenylation of plastoquinone, and is also an intermediate in the synthesis of C_20_ GGPP. A single predicted type II GGPPS catalyzes the formation of this C_20_ precursor from IPP and DMAPP in the plastid by sequentially adding C_5_ units to the growing prenyl chain [2]. C_10_ GPP and C_15_ FPP are generated as intermediates, which have not been reported to be released from the enzyme as side products. However, a type II GGPP synthase from another green microalga, *Haematococcus lacustris*, exhibited weak product promiscuity for FPP in vitro [19], and the same was reported for a cyanobacterial GGPPS [20]. Therefore, plastidic FPP may be rapidly displaced and re-bound from the GGPPS active site as a reaction species, and FPP leakage from the GGPPS may represent a remnant of a prokaryotic evolutionary ancestor. FPP import from the cytosol to the chloroplast may also explain the presence of this precursor, but import of prenyl diphosphates into plant chloroplasts has been excluded previously [21].

### C_5_ IPP and DMAPP are more readily available in the algal chloroplast than in the cytosol

It was expected that isoprenoid turnover and flux towards prenyl precursors is higher in the chloroplast due to operation of the native MEP pathway in this compartment. Co-expression of *Sc*ERG20 and *Ag*BS in the chloroplast increased bisabolene production ~ 16-fold over the parental strain (Fig. 2), whereas co-expression of the native *Cr*FPPS and *Ag*BS in the cytosol did not alter the yield (Additional file 11: Fig. S1). Co-overexpression of an FPPS and the patchoulol synthase in tobacco plastids also improved patchoulol production several-fold [22]. This indicates a large plastidic pool of IPP and DMAPP, which is freely available in this compartment. Fusion of a terpene synthase to *Sc*ERG20(F96C) was previously used to boost diterpenoid product formation from the algal chloroplast due to channeling from C_5_ prenyl precursors via higher prenyl intermediates to the final product [6]. We therefore hypothesized that the *Ag*BS alone and in fusion with *Sc*ERG20 could serve as a reporter to estimate the abundances of FPP, IPP, and DMAPP in the cytosol and the chloroplast. Targeting of the *Ag*BS-mVenus-*Sc*ERG20 fusion (construct x) to the chloroplast indeed resulted in 25-fold improved bisabolene yields compared to the reference construct (vector i) (Fig. 3). Exchange of the *Sc*ERG20 fusion partner with the native *Cr*FPPS led to a less pronounced enhancement (ninefold), which may result from a lower activity of this enzyme compared to the yeast ERG20. There is strong evidence that the C_5_ precursor pool in the chloroplast is much larger than C_5_ in the cytosol. Although FPP was observed in the chloroplast by the capacity to produce bisabolene, it was not much greater than that natively available in the cytosol (Fig. 3). Nevertheless, the biosynthetic potential for sesquiterpenoid production from the plastid is markedly improved when FPPS is co-expressed to convert C_5_ precursors to FPP. A large supply of plastidic C_5_ prenyl precursors may reflect the natural ability of green algae to adapt to highly variable light conditions [23].

### The unique nature of green algal FPP metabolism

A direct comparison of sesquiterpene production by the same enzyme targeted to cytoplasm or chloroplast may be hindered by several factors like differing chemical environments (pH, Mg^2+^ concentrations), differing substrate concentrations, and substrate channeling effects of multi-enzyme complexes. However, we observed heterologous bisabolene production at comparable low levels from *Ag*BS targeted to either cellular compartment. The question, therefore, is where does the cytoplasmic FPP come from if overexpression of an FPPS in the cytoplasm does not increase sesquiterpene yields (Fig. 3)? This becomes more intriguing when one considers the specific localization of the *Cr*FPPS, in a halo around the algal nucleus (Fig. 3), a region where the ER-nuclear association is expected to be found. This localization could enable efficient channeling of FPP to the squalene synthase (*Cr*SQS) anchored in the ER membrane in proximity (Fig. 3, [15]), and would prioritize FPP for sterol synthesis. Indeed, knockdown of *Cr*SQS results in increased sesquiterpene yields in the algal cytoplasm [8]. Nevertheless, the origin of DMAPP and IPP in this situation seems enigmatic. It was hypothesized that targeting of the *Ag*BS to the vicinity of these enzymes would also channel FPP to the active site of this sesquiterpene synthase and enhance bisabolene yields. Therefore, the *Ag*BS-mVenus construct was fused to *Cr*FPPS and the transmembrane domain of *Cr*SQS. Although the desired targeting effect was achieved (Fig. 3), bisabolene yields did not improve and were actually lower than the *Ag*BS alone targeted to the cytosol. Considering these observations, two metabolic modes of C_5_ and C_15_ prenyl unit generation and transport (or a mixture of both) may be possible:

(i) IPP and DMAPP are exported from the chloroplast at low rates in specific connection to the site of FPP synthesis. The generation of FPP by the native, nucleus envelope-associated *Cr*FPPS could be highly efficient, hence cytosolic IPP and DMAPP concentrations are low enough to not yield improved bisabolene yields with FPPS fusion proteins. The transfer of FPP to the *Cr*SQS could be inefficient, and FPP may leak into the cytosol, resulting in a freely available metabolic pool. This may enable uptake into mitochondria and supply protein prenylation and dolichol production. There are two IPP isomerases (Cre08.g381800, Cre11.g467544, Phytozome v5.6) in the genome of *C. reinhardtii*, one of which may be required to maintain stoichiometries of IPP and DMAPP that match the differing substrate demands for plastidic GGPP and cytosolic FPP synthesis, and suggests a significant role of the two C_5_ precursors in both compartments.

On the contrary (ii), FPP could be exported from the chloroplast, potentially by an ancient IPP/DMAPP transporter. In this case, the FPPS would play a minimal role in FPP synthesis, which would take place in the chloroplast. FPP exported from the chloroplast could assist cytosolic formation of various C_15_-derived terpenoids, e.g. sterols. In both situations, whether exported or not, the small amount of plastidic FPP could be a promiscuous by-product of GGPP synthesis, and possibly also assist plastidic SPP formation. In this case, FPP leakage from the GGPPS may represent a remnant of a prokaryotic evolutionary ancestor [20]. However, it is questionable if the plastidic GGPPS is an FPP-leaking enzyme like in *H. lacustris* [19] and simultaneously the major source of (exported) FPP. Evidence against this was the finding that *Cr*GGPPS knock-down resulted in increased FPP supply to yield improved bisabolene titers in the cytosol [8], indicating a more dominant role of IPP/DMAPP export. Indeed, here *Cr*FPPS was active in conversion of IPP and DMAPP into C_15_ substrate in the algal chloroplast (Fig. 3). However, directing *Ag*BS to the site of *Cr*FPPS in cytoplasmic fusions had no improvement over the *Ag*BS alone, suggesting a lack of free C_5_ precursor in this location. It is likely that *C. reinhardtii*, like higher plants, regulates cytosolic IPP/DMAPP abundance using an isopentenyl phosphate kinase (IPK) and a Nudix superfamily hydrolase as metabolic valve [9, 24]. A putative IPK is encoded in the genome of *C. reinhardtii* (*Cre13.g579800*, Uniprot A8HTR6), and could function together with a Nudix superfamily hydrolase (yet to be identified) which may explain low cytosolic C_5_ precursor abundances in this organism.

A mixture of both situations (i & ii) is possible, and in line with this, plant chloroplasts were found to be capable of FPP export by a yet unknown mechanism, which was determined to be slower than IPP export [21]. Higher plants and green algae share many metabolic features, but algae differ as they have lost the cytoplasmic/peroxisomal mevalonate pathway and the respective ability of this pathway in higher plants to provide FPP in various compartments: while Chlorophycean algae possess a single annotated FPPS as described here, higher plants have multiple isoforms to generate FPP pools in mitochondria, peroxisomes, and likely in the cytosol relatively independently [25].

### Candidates for a prenyl transporter

Members of all eukaryotic kingdoms require prenyl unit exchange across lipid bilayer membranes. Although export of C_5_, C_10_, and C_15_ isoprenoid precursors from higher plant chloroplasts has been reported [21, 26], its exact mechanism is unclear. The uniqueness and apparent controlled specificity of algal metabolic architecture may require a transporter to facilitate the observed C_15_ FPP availability in the cytoplasm of the alga. We sought to use bioinformatics to assist in finding previously uncharacterized transporters that may play a role in this. By narrowing putative transporters down to plastid membrane-localized candidates without known substrates and uptake function, we were able to find one protein sequence (UniProt A0A2K3DRV6, Phytozome Cre05.g232751) annotated as a multidrug efflux transporter. A CLiP mutant with an insertion in the coding sequence is available [27], indicating that this gene could be non-essential. It was surprising to us that only one candidate could be found, which has no significant similarity to transporters in other organisms except uncharacterized/hypothetical proteins in other algae and bacteria. Perhaps such a protein enabled the transport of amphipathic compounds like FPP and the loss of the MVA pathway in many algae species? However, this finding is preliminary and will need to be followed up by in vivo localization, substrate testing and possibly its expression in plants concomitant with MVA pathway knockout. Such investigations are, however, beyond the scope of this report.

### *Ag*BS catalytic efficiency limits abundant FPP conversion to bisabolene

Only marginal yield improvements were achieved through gene loading strategies to accumulate *Ag*BS copies in the chloroplast in an ERG20 co-overexpression background (Fig. 4A) or to obtain more *Ag*BS-reporter-ERG20 fusion protein (Fig. 4B). Bisabolene yields previously obtained from the cytosol after *Cr*SQS knockdown [8] could also not be matched or out-competed with our strategy of tapping plastidic prenyl pools for sesquiterpene synthesis (Figs. 4 and 5). We expected that the chloroplast bears higher potential for this challenge, but this capacity can likely not be exhausted at this juncture. Green algal GGPP synthases were shown to convert not only C_5_ prenyl pyrophosphate but also C_15_ FPP to its C_20_ product [19], and it is possible that excess FPP in the chloroplast is rapidly scavenged by this enzyme in *C. reinhardtii* while being converted to sesquiterpene products at comparably low flux by catalytically inefficient sesquiterpene synthases like the *Ag*BS. Perhaps a higher residence time of FPP due to SQS knockdown in the cytoplasm led to the increased titers observed in our previous work [8]. A homologous algal GGPPS from *H. pluvialis* exhibited higher affinity (*K*_*M*_ = 19 μM) towards FPP in vitro [19] than the *Ag*BS with a *K*_*M*_ of 49.5 ± 6.3 μM [28]. An imbalance between rapid FPP generation by the *Sc*ERG20 FPPS and subsequent slow conversion to bisabolene by the *Ag*BS may be responsible for this observation. Extra FPP produced by the *Sc*ERG20 is likely processed to GGPP and pigments rapidly by the native cellular machinery.

It could also be possible that the freely accessible IPP and DMAPP pools of the chloroplast were totally depleted by robust *Sc*ERG20 activity, leading to a limited increase in bisabolene titers in cell lines transformed with multiple *Ag*BS-reporter-*Sc*ERG20 fusion constructs. However, no apparent effect on cellular pigment levels was observed, suggesting that the IPP and DMAPP pool was not yet depleted. Gene loading strategies like the one applied here may be obsolete in the near future due to improved transgene expression with optimized genetic tools such as synthetic promoters which can generate more recombinant protein products from single transformation events [16].

### Light–dark cycling encourages heterologous isoprenoid production from the chloroplast

We have previously demonstrated that L:D illumination cycles and photoautotrophic conditions promote high production rates for heterologous diterpenoids (C_20_) from the chloroplast [6]. In this work, elevated specific productivities of the sesquiterpene bisabolene could also be observed in these conditions (Fig. 5C), although diterpenoid production rates [6] could not be matched likely due to the low *Ag*BS catalytic efficiency [28]. Prenyl precursors might be more readily available for heterologous terpene synthases in the dark as the pull to their roles in light-processes, synthesis of pigments and electron transport chain compounds, is stopped. Green algae like *C. reinhardtii* are adapted to a broad range of fluctuating light intensities and therefore can rapidly react to sudden illumination upon dark phases. A large pool of prenyl substrates held in the dark may support this ability. Simultaneous provision of CO_2_ in excess could further stimulate flux towards isoprenoids due to NAB1-mediated de-repression of LHCII protein accumulation, which affects both bound chlorophylls as well as carotenoids representing a metabolic pull in these conditions [29].

## Conclusions

With this study, we aimed to elucidate and exploit the compartmentalization of FPP metabolism in a green algal host by using the sesquiterpene product (*E*)-α-bisabolene as a reporter. Many details remain elusive, especially which prenyl precursors (C_5_ and/or C_15_, di- and/or monophosphates) are exported from the chloroplast, which transporter is involved, and how FPP is channeled towards distinct enzymes like the SQS or to other organelles such as mitochondria. However, our findings that FPP is available for sesquiterpenoid production in the chloroplast and that highly abundant IPP and DMAPP precursors can be tapped in this compartment will further progress engineering efforts of efficient sesquiterpenoid generation from algal hosts like *Chlamydomonas*. Compared to other strategies implemented in *C. reinhardtii* to produce bisabolene from the cytosol, expressing the *Ag*BS fused to an FPPS like *Sc*ERG20 to the chloroplast as in this work was very effective (Table 1).

**Table 1:** Effectiveness of metabolic engineering strategies for bisabolene production from *C. reinhardtii* as a production host

Engineering strategy	Cellular compartment	No. of successive transformations	Max. bisabolene titer (mg L^−1^)	Refs.
*Ag*BS expression	Cytosol	1	0.4	[8]
Sequential *Ag*BS transformations + SQS knock-down	Cytosol	4	4.8	[8]
Use of optimized promoter-terminator combination	Cytosol	1	2.5	[16]
Co-expression of *Ag*BS and FPPS	Chloroplast	2	3.2	This study
*Ag*BS and FPPS fusion	Chloroplast	1	4.3	This study
Sequential *Ag*BS-FPPS fusion transformations	Chloroplast	4	8.4	This study

During the time of the investigations presented in this work, genetic tools for *C. reinhardtii* have been further optimized [11, 16]. Using an optimized promoter-terminator combination resulted in more than fourfold higher bisabolene titers [16] compared to the common promoter-terminator combination used in a previous publication [8] (Table 1). In the future, improved transgene expression tools should allow us to overcome limitations of terpene synthase titer imposed by low expression from heterologous transgenes in algae. Higher expression of recombinant enzymes in fewer transformation rounds would allow combinations of the most promising engineering strategies in one production strain without exhausting selection markers. We suggest that simultaneous sesquiterpene production from the chloroplast and the cytosol [8] will be possible when using these optimized genetic tools and will enhance algal sesquiterpenoid production capacities. Future engineering efforts should involve the AβSAP(i)-FDX1 and PSAD-PSAD promoter-terminator combinations [16] to maximize sesquiterpene synthase and sesquiterpene synthase-FPPS enzyme accumulation in the cytosol and the chloroplast, respectively. Further improvements with respect to yield, titer, and rate can be expected from combining these engineering steps with de-regulation of the MEP pathway; by metabolic push (i.e. overexpression of 1-deoxy-d-xylulose-5-phosphate synthase) [6], or metabolic pull through modification of carotenoid biosynthesis (i.e. lycopene cyclase [30, 31]). Importantly, the strategies developed here can be applied to the production of any sesquiterpenoid, including compounds of higher value with more realistic economic potential than the biofuel precursor bisabolene. Apart from engineering cell factory performance, titers and volumetric culture productivities can additionally be enhanced by high cell density cultivation as recently demonstrated [32]. If a prenyl transporter can be identified, this may be leveraged for improving export to a cytosolic sesquiterpene generating pathway. Nevertheless, the possibility of exploiting isoprenoid metabolism in the chloroplast for sesquiterpene generation investigated in this work will be valuable for future metabolic engineering efforts to establish this and other microalgal strains as efficient sesquiterpene generating cell factories.

## Methods

### *C. reinhardtii* strains and cultivations

*Chlamydomonas reinhardtii* strain UVM4 [33] was maintained in TAP medium [34] at 150–200 µmol photons m^−2^ s^−1^ light intensity in shake flasks or on agar plates. Scale-up cultures were gently stirred in 1000 mL Erlenmeyer flasks containing 380 mL media and 20 mL dodecane overlay. Illumination was performed with 100 µmol photons m^−2^ s^−1^ constant light or 16:8 h light:dark cycles using air or 3% CO_2_ surface gassing as indicated. Cultivations were performed in TA2P or T2P media differing in the presence or absence of acetate [8].

### Plasmid construction, transformation, screening, and imaging of transformants

Cloning was performed with Thermo Scientific FastDigest restriction enzymes and the Rapid DNA Dephos & Ligation Kit (Roche) according to manufacturers’ protocols. Q5 high fidelity polymerase (New England Biolabs) was used to conduct PCRs following manufacturer’s protocols, with primers listed in Additional file 1. All plasmid sequences were confirmed by sequencing (Sequencing Core Facility, CeBiTec, Bielefeld University, Germany). Plasmids were maintained in *Escherichia coli* DH5α.

Nucleotide sequences (*Ag*BS (UniProt ID: O81086) [8], *Cr*FPPS (Uniprot ID: A8IX41), *Saccharomyces cerevisiae* ERG20 (UniProt ID: P08524) [7] were codon optimized by back translating the amino acid sequence and synthesized (Genscript) with enhancing introns as previously reported [7, 10, 35, 36]. Compatible restriction endonuclease cut sites were incorporated for insertion into the pOptimized_2.0 (pOpt_2.0) vector system [8]. The *Ag*Bs and *Sc*ERG20 were cloned to create fusions with mVenus, mCerulean3, or mRuby2 fluorescent protein (Y/C/RFP) reporters. Targeting to the chloroplast was achieved with the photosystem I reaction center subunit II (PsaD) signal peptide as described [6]. All constructs are listed in Table 2.

**Table 2:** Genetic constructs used in this study

Construct name	Vector number	Antibiotic resistance in *C. reinhardtii*	Gene length with introns from start codon to stop (bp)	CDS (bp)	RBCS2 i1 copies	Protein length (aa)	Predicted molecular weight (kDA)	Accession No. (NCBI)/reference
pOpt2_PsaD-AgBS-mVenus_Paro	i	Paromomycin	4701	3357	7	1118	127.4	This work
pOpt2_PsaD-mCerulean3_Hyg	ii	Hygromycin B	1395	921	1	306	50.0	[6]
pOpt2_PsaD-ERG20-mCerulean3_Hyg	iii	Hygromycin B	2753	1989	3	662	74.8	This work
pOpt2_PsaD-mCerulean3-ERG20_Hyg*	iv	Hygromycin B	2711	1947	3	648	73.2	This work
pOpt2_AgBS-mVenus_Paro	v	Paromomycin	4605	3261	7	1086	124.2	[8]
pOpt2_AgBS-mVenus-ERG20_Paro*	vi	Paromomycin	6014	4380	9	1459	166.5	This work
pOpt2_AgBS-mVenus-CrFPPS_Paro*	vii	Paromomycin	6084	4305	10	1434	163.0	This work
pOpt2_AgBS-mVenus-CrSQS_TMD__Paro	viii	Paromomycin	4839	3495	7	1164	132.8	This work
pOpt2_PsaD-AgBS-mVenus-CrFPPS_Paro*	ix	Paromomycin	6192	4413	10	1470	166.8	This work
pOpt2_PsaD-AgBS-mVenus-ERG20_Paro*	x	Paromomycin	6014	4380	9	1459	166.5	This work
pOpt2_PsaD-mRuby2_Ble	xi	Zeocin	1389	915	1	304	33.9	This work
pOpt2_PsaD-AgBS-mRuby2_Ble	xii	Zeocin	4707	3363	7	1120	127.5	This work
pOpt2_PsaD-aadA2_MinS	xiii	Spectinomycin	1467	993	1	330	36.7	[6]
pOpt2_PsaD-AgBS-aadA2_MinS	xiv	Spectinomycin	4785	3441	7	1146	130.3	This work
pOpt2_PsaD-AgBS-mCerulean3-ERG20_Hyg*	xv	Hygromycin B	6029	4395	9	1464	166.8	This work
pOpt2_PsaD-AgBS-mRuby2-ERG20_Ble*	xvi	Zeocin	6023	4389	9	1462	166.6	This work
pOpt2_PsaD-AgBS-aadA2-ERG20_MinS*	xvii	Spectinomycin	6101	4467	9	1488	169.5	This work
pOpt2_mCerulean3_Hyg	xviii	Hygromycin B	1287	813	1	270	30.4	[8]
pOpt2_CrFPPS-mCerulean3_Hyg	xix	Hygromycin B	2820	1911	4	636	71.3	This work
pOpt2_mCerulean3-CrFPPS_Hyg*	xx	Hygromycin B	2778	1869	4	622	69.6	This work
		**UniProt ID**						
*Ag*Bs alone		O81086	3318	2448	6	816	93.6	MG052654 [8]
ERG20 alone		P08524	1346	1056	2	352	40.5	KX097889 [7]
*Cr*FPPS alone		A8IX41	1515	1080	3	360	40.5	This work

^*^Constructs with N-terminal FPPS sequences were modified to contain a stop codon 5’ of the StrepII tag

1 copy of the RBCS2 intron 1 (i1) is found within the HSP70-RBCS2 promoter. In addition, all mVenus-, mCerulean3-, or mRuby2 linked constructs contain the RBCS2i2 intron in the fluorescent reporter as in [38]

Glass bead mediated transformations were conducted as previously described [37]. Positive transformants were selected on TAP agar plates supplemented with suitable antibiotics at 10 mg L^−1^ (paromomycin), 20 mg L^−1^ (hygromycin B), 15 mg L^−1^ (zeocin), or 200 mg L^−1^ (spectinomycin). Target construct expression was identified with fluorescence microscopy for YFP, CFP, or RFP fluorescence signal as reported [38]. Full-length expression of all enzyme-reporter fusions was confirmed by SDS PAGE and Western blotting using α-GFP-HRP linked antibody (Life technologies), α-StrepII tag-HRP linked antibody (IBA Lifesciences), or α-aadA (Agrisera, AS09 580) and corresponding anti-rabbit-HRP secondary antibody. Fluorescence microscopy was conducted as described [7].

### Capture and analysis of bisabolene

Bisabolene production was analyzed for each transgene construct with three independent cell lines in biological triplicates by two-phase cultivation with a dodecane overlay (4.5 mL culture and 0.5 mL overlay) in clear 6-well plates and subsequent gas chromatography mass spectroscopy (GC–MS) analysis using a TraceGC gas chromatograph and PolarisQ ion trap mass spectrometer equipped with a AS 3000 autosampler (Thermo Scientific, Germany) and a 30-m 0.25-mm VF-5 ms column coated with 0.25 mm of 5% diphenyl and 95% dimethylsiloxane (Varian GmbH, Darmstadt, Germany) as previously described [6–8]. Bisabolene peak areas were determined using total ion current (TIC) and normalized to internal standard α-humulene for quantification against standard calibration curve (Additional files 2, 3, 4) as previously described [8].

### Identification of putative prenyl diphosphate transporters

All annotated proteins for *C. reinhardtii* CC-503 (DoE assembly version 5.5) were downloaded from the NCBI ftp site (ftp://ftp.ncbi.nlm.nih.gov/genomes/all/GCA/000/002/595/GCA_000002595.3_Chlamydomonas_reinhardtii_v5.5O) and all annotated plastidial proteins (https://www.ncbi.nlm.nih.gov/nuccore/FJ423446.1) were extracted from the NCBI website as a fasta file of protein sequences. Protein sequences were then analyzed with the membrane transporter analysis tool TransAAP [39] (http://www.membranetransport.org/transportDB2), to identify putative transporters. A local version of this was run which allows a more permissive inclusion of transporters than the publicly available version, to maximise the chances of characterising novel transporters (Additional file 5, Additional file 6).

To determine the subcellular localization of the potential transporter candidates for farnesyl diphosphate transport across the plastid membrane, DeepLoc 1.0 was utilized [40] (https://services.healthtech.dtu.dk/service.php?DeepLoc-1.0) on the 'fast' setting (BLOSUM62 protein encoding). As DeepLoc is relatively slow, and as a web service, has restrictions on submission size, only protein sequences identified by TMHMM 2.0 [41] as having at least one transmembrane sequence (TMS, a feature of the TransAAP software), were analyzed (Additional file 7). This required subsetting of the sequence file, and elimination of a small number of sequences that exceeded the 6000 aa limit, leaving 3540 protein sequences for analysis (Additional file 8). Based on their size, and number of TMS, the omitted sequences were unlikely to be candidates for prenyltransferases or relevant transporters.

The full set of proteins that potentially localized to the plastid membrane totaled 182 (Additional file 9). In order to identify potential farnesyl transferases, the hmmscan utility from the HMMER suite of programs (version 3.3.2) (www.hmmer.org) [42] was used to compare the set of 182 proteins that localized to the plastid membrane to the Pfam UbiA HMM (https://pfam.xfam.org/family/UbiA) PF01040 (this PFAM represents the characteristic domain of the prenyl transferases). Two sequences had a significant match, PNW82555.1 (Phytozome Cre06.g283750) and PNW82799.1 (Phytozome Cre06.g294750), but both have annotated functions that would exclude them as candidates for prenyltransferase activity. Of 182 plastid membrane proteins, 44 were also designated as transporters by TransAAP (Additional file 10). This set of 44 was manually curated to eliminate transporters unlikely to efflux substrates based on their association with uptake, or clearly having a specific substrate as suggested by the TransAAP designation or closest matches in BLAST searches. Similarly, the 21 putative transporters identified by TransAAP belonging to the plastid genome were examined, with all of them having clearly defined functions that are not farnesyl diphosphate transport. This left one protein sequence, PNW83272.1 (UniProt A0A2K3DRV6, Phytozome Cre05.g232751), which TransAAP annotated as multidrug efflux (MFS) transporter, which can be considered a generic description to suggest the substrate is an amphipathic molecule.

## Supplementary Information

**Additional file 1. **A list of oligonucleotides used for cloning of genetic constructs as listed in Table 2.

**Additional file 2. **Calculations for bisabolene standard quantification.

**Additional file 3. **Calculation of bisabolene titers, yields, and rates for all mutants created in this work as shown in Additional file 11: Fig. S1 and Figs. 2, 3, and 4.

**Additional file 4. **Cell densities, CDW, and calculation of bisabolene titers and production rates for scale-up cultivations of cell line containing constructs x + xv + xvi as shown in Fig. 5.

**Additional file 5. **All transporters annotated by TransAAP, with duplications removed.

**Additional file 6. **Transporters annotated on the chloroplast genome by TransAAP

**Additional file 7. **3557 Protein sequences identified as having one or more putative TMS by TMHMM.

**Additional file 8. **DeepLoc assigned localization results for the protein sequences in ‘list_of_chlamy_with_tms’. 17 sequences were not able to be processed due to exceeding the size limit for DeepLoc (6000 aa), but based on their length and number of TMs they were extremely unlike to be candidates of interest.

**Additional file 9. **This file contain the names of all the protein sequences that were identified by DeepLoc, the complete list being in Additional file 9, as being localized to the chloroplast membrane.

**Additional file 10. **This contains the 44 protein sequences that were both annotated as transporters by TransAAP and localized to the chloroplast membrane by DeepLoc.

**Additional file 11: Figure S1.** Attempts to increase flux towards cytosolic FPP by *Cr*FPPS accumulation. Different orientations with the mCerulean3 reporter and co-expression of *Ag*BS and corresponding bisabolene yields (fg cell^−1^) are depicted (constructs indicated on the left). Error bars represent 95% confidence intervals. **Figure S2.** Detection of full-length protein products of 3 independent cell lines transformed with various constructs. Each cell line is denoted by individual numbers, constructs are specified at the top, and corresponding bisabolene yields are depicted in Fig. 2. Total cellular proteins were run on a 10% SDS PAGE gel and subjected to Western blotting with α-GFP antibody. M marker (PageRuler prestained protein ladder, ThermoFisher Scientific). WT parental strain UVM4. P positive control expressing *Ag*BS-mVenus, *Ag*BS-mCerulean3, and *Ag*BS-mRuby2 to the cytosol. Positive control and ERG20 protein products show previously observed pattern of protein breakage in the reporter [7]. Predicted protein sizes are: ~ 124.0 kDa for *Ag*BS-mVenus, ~ 70.2 kDa for ERG20-mCerulean3, and ~ 67.7 kDa for mCerulean3-ERG20 which lacks a StrepII tag on its C terminus. **Figure S3.** Detection of full-length protein products of 3 independent cell lines transformed with various constructs. Each cell line is denoted by individual numbers, constructs are specified at the top, and corresponding bisabolene yields are depicted in Fig. 3. Total cellular proteins were run on a 10% SDS PAGE gel and subjected to Western blotting with α-GFP antibody. M marker (PageRuler prestained protein plus ladder, ThermoFisher Scientific). WT parental strain UVM4. **Y** mVenus expressed to the cytosol. Predicted protein sizes are: ~ 124.0 kDa for *Ag*BS-mVenus, ~ 161.9 kDa for *Ag*BS-mVenus-ERG20. Ponceau S stain is shown as loading control on the nitrocellulose membrane. Signal for construct vi, strain #3 was below detection limit, but bisabolene could be detected in GC–MS measurements, indicating full-length expression. **Figure S4.** Detection of full-length protein products of 3 independent cell lines transformed with various constructs. Each cell line is denoted by individual numbers, constructs are specified at the top, and corresponding bisabolene yields are depicted in Fig. 4. Total cellular proteins were run on a 10% SDS PAGE gel and subjected to Western blotting with α-GFP and α-StrepII antibodies. M marker (PageRuler prestained protein ladder, ThermoFisher Scientific). WT parental strain UVM4. P positive control expressing *Ag*BS-mVenus, AgBS-mCerulean3, and AgBS-mRuby2 to the cytosol. Positive control and ERG20 protein products show previously observed pattern of protein breakage in the reporter [7]. Predicted protein sizes are: ~ 124.0 kDa for *Ag*BS-mVenus, ~ 123.1 kDa for *Ag*BS-mCerulean3, ~ 120.9 kDa for *Ag*BS-mRuby2, ~ 123.7 kDa for *Ag*BS-aadA, ~ 67.7 kDa for mCerulean3-ERG20 which lacks a StrepII tag on its C terminus, ~ 161.9 kDa for *Ag*BS-mVenus-ERG20, and ~ 162.2 kDa for *Ag*BS-mCerulean3-ERG20. **Figure S5.** Detection of full-length protein products of 3 independent cell lines transformed with various constructs. Each cell line is denoted by individual numbers, constructs are specified at the top, and corresponding bisabolene yields are depicted in Fig. 3 and Additional file 11 Fig. S1. Total cellular proteins were run on a 10% SDS PAGE gel and subjected to Western blotting with α-GFP antibodies. M marker (PageRuler prestained protein ladder, ThermoFisher Scientific). WT parental strain UVM4. *Cr*FPPS protein products show previously observed pattern of protein breakage in the reporter [7]. Predicted protein sizes are: ~ 160.9 kDa for *Ag*BS-mVenus-*Cr*FPPS, ~ 124.0 kDa for *Ag*BS-mVenus, ~ 68.5 kDa for *Cr*FPPS-mCerulean3, and ~ 67.0 kDa for mCerulean3-*Cr*FPPS which lacks a StrepII tag on its C terminus. **Figure S6.** Calibration line and correspondent equation for bisabolene quantification. GC/MS peak areas were normalized to the internal standard peak area, and plotted against the bisabolene concentration in dodecane.

## Data Availability

All data generated or analyzed during this study are included in this published article and its additional data files.

## Abbreviations

*Ag*Bs: *Abies grandis* Bisabolene synthase
CDW: Cell dry weight
IPP: Isopentenyl pyrophosphate
DMAPP: Dimethylallyl pyrophosphate
FPP: Farnesyl pyrophosphate
GGPP: Geranylgeranyl pyrophosphate
TAP: Tris Acetate Phosphate medium
TA2P: TAP medium with 2X phosphate content
T2P: TAP medium without acetate and 2X phosphate content
YFP: mVenus yellow fluorescent protein
CFP: mCerulean3 cyan fluorescent protein
RFP: mRuby2 red fluorescent protein
PQ: Plastoquinone
UQ: Ubiquinone
PFT: Protein farnesyl transferase
FPPS: Farnesyl pyrophosphate synthase
GGPPS: Geranylgeranyl pyrophosphate synthase
SQS: Squalene synthase

## References

[1] Gershenzon J, Dudareva N (2007). The function of terpene natural products in the natural world. Nat Chem Biol.

[2] Lohr M, Schwender J, Polle JEW (2012). Isoprenoid biosynthesis in eukaryotic phototrophs: a spotlight on algae. Plant Sci.

[3] Misawa N (2011). Pathway engineering for functional isoprenoids. Curr Opin Biotechnol.

[4] Carruthers DN, Lee TS (2021). Diversifying isoprenoid platforms via atypical carbon substrates and non-model microorganisms. Front Microbiol.

[5] Lauersen KJ (2019). Eukaryotic microalgae as hosts for light-driven heterologous isoprenoid production. Planta.

[6] Lauersen KJ, Wichmann J, Baier T, Kampranis SC, Pateraki I, Møller BL (2018). Phototrophic production of heterologous diterpenoids and a hydroxy-functionalized derivative from *Chlamydomonas reinhardtii*. Metab Eng.

[7] Lauersen KJ, Baier T, Wichmann J, Wördenweber R, Mussgnug JH, Hübner W (2016). Efficient phototrophic production of a high-value sesquiterpenoid from the eukaryotic microalga *Chlamydomonas reinhardtii*. Metab Eng.

[8] Wichmann J, Baier T, Wentnagel E, Lauersen KJ, Kruse O (2018). Tailored carbon partitioning for phototrophic production of (*E*)-α-bisabolene from the green microalga *Chlamydomonas reinhardtii*. Metab Eng.

[9] Henry LK, Thomas ST, Widhalm JR, Lynch JH, Davis TC, Kessler SA (2018). Contribution of isopentenyl phosphate to plant terpenoid metabolism. Nat Plants.

[10] Baier T, Wichmann J, Kruse O, Lauersen KJ (2018). Intron-containing algal transgenes mediate efficient recombinant gene expression in the green microalga *Chlamydomonas reinhardtii*. Nucleic Acids Res.

[11] Baier T, Jacobebbinghaus N, Einhaus A, Lauersen KJ, Kruse O (2020). Introns mediate post-transcriptional enhancement of nuclear gene expression in the green microalga *Chlamydomonas reinhardtii*. PLoS Genet.

[12] Perozeni F, Cazzaniga S, Baier T, Zanoni F, Zoccatelli G, Lauersen KJ (2020). Turning a green alga red: engineering astaxanthin biosynthesis by intragenic pseudogene revival in *Chlamydomonas reinhardtii*. Plant Biotechnol J.

[13] Bohlmann J, Crock J, Jetter R, Croteau R (1998). Terpenoid-based defenses in conifers: cDNA cloning, characterization, and functional expression of wound-inducible (*E*)-alpha-bisabolene synthase from grand fir (*Abies grandis*). Proc Natl Acad Sci USA.

[14] Ignea C, Trikka FA, Nikolaidis AK, Georgantea P, Ioannou E, Loupassaki S (2015). Efficient diterpene production in yeast by engineering Erg20p into a geranylgeranyl diphosphate synthase. Metab Eng.

[15] Kajikawa M, Kinohira S, Ando A, Shimoyama M, Kato M, Fukuzawa H (2015). Accumulation of squalene in a microalga *Chlamydomonas reinhardtii* by genetic modification of squalene synthase and squalene epoxidase genes. PLoS ONE.

[16] Einhaus A, Baier T, Rosenstengel M, Freudenberg RA, Kruse O (2021). Rational promoter engineering enables robust terpene production in microalgae. ACS Synth Biol.

[17] Hsu SC, Browne DR, Tatli M, Devarenne TP, Stern DB (2019). N-terminal sequences affect expression of triterpene biosynthesis enzymes in *Chlamydomonas* chloroplasts. Algal Res.

[18] Zhan X, Zhang Y-H, Chen D-F, Simonsen HT (2014). Metabolic engineering of the moss *Physcomitrella patens* to produce the sesquiterpenoids patchoulol and α/β-santalene. Front Plant Sci.

[19] Lao YM, Jin H, Zhou J, Zhang HJ, Zhu XS, Cai ZH (2018). A novel hydrolytic activity of tri-functional geranylgeranyl pyrophosphate synthase in *Haematococcus pluvialis*. Plant Cell Physiol.

[20] Satta A, Esquirol L, Ebert BE, Newman J, Peat TS, Plan M (2022). Molecular characterization of cyanobacterial short-chain prenyltransferases and discovery of a novel GGPP phosphatase. FEBS J.

[21] Bick JA, Lange BM (2003). Metabolic cross talk between cytosolic and plastidial pathways of isoprenoid biosynthesis: unidirectional transport of intermediates across the chloroplast envelope membrane. Arch Biochem Biophys.

[22] Wu S, Schalk M, Clark A, Miles RB, Coates R, Chappell J (2006). Redirection of cytosolic or plastidic isoprenoid precursors elevates terpene production in plants. Nat Biotechnol.

[23] Wobbe L, Bassi R, Kruse O (2016). Multi-level light capture control in plants and green algae. Trends Plant Sci.

[24] Henry LK, Gutensohn M, Thomas ST, Noel JP, Dudareva N (2015). Orthologs of the archaeal isopentenyl phosphate kinase regulate terpenoid production in plants. Proc Natl Acad Sci.

[25] Thabet I, Guirimand G, Courdavault V, Papon N, Godet S, Dutilleul C (2011). The subcellular localization of periwinkle farnesyl diphosphate synthase provides insight into the role of peroxisome in isoprenoid biosynthesis. J Plant Physiol.

[26] Flügge UI, Gao W (2005). Transport of isoprenoid intermediates across chloroplast envelope membranes. Plant Biol.

[27] Li X, Patena W, Fauser F, Jinkerson RE, Saroussi S, Meyer MT (2019). A genome-wide algal mutant library and functional screen identifies genes required for eukaryotic photosynthesis. Nat Genet.

[28] McAndrew RP, Peralta-Yahya PP, Degiovanni A, Pereira JH, Hadi MZ, Keasling JD (2011). Structure of a three-domain sesquiterpene synthase: a prospective target for advanced biofuels production. Structure.

[29] Berger H, Blifernez-Klassen O, Ballottari M, Bassi R, Wobbe L, Kruse O (2014). Integration of carbon assimilation modes with photosynthetic light capture in the green alga *Chlamydomonas reinhardtii*. Mol Plant.

[30] Moreno JC, Mi J, Agrawal S, Kössler S, Turecková V (2020). Expression of a carotenogenic gene allows faster biomass production by redesigning plant architecture and improving photosynthetic efficiency in tobacco. Plant J.

[31] Kössler S, Armarego-Marriott T, Tarkowská D, Turečková V, Agrawal S, Mi J (2021). Lycopene β-cyclase expression influences plant physiology, development, and metabolism in tobacco plants. J Exp Bot.

[32] Freudenberg RA, Baier T, Einhaus A, Wobbe L, Kruse O (2021). High cell density cultivation enables efficient and sustainable recombinant polyamine production in the microalga *Chlamydomonas reinhardtii*. Bioresour Technol.

[33] Neupert J, Karcher D, Bock R (2009). Generation of *Chlamydomonas* strains that efficiently express nuclear transgenes. Plant J.

[34] Gorman DS, Levine RP (1965). Cytochrome f and plastocyanin: their sequence in the photosynthetic electron transport chain of *Chlamydomonas reinhardi*. Proc Natl Acad Sci.

[35] Lauersen KJ, Berger H, Mussgnug JH, Kruse O (2013). Efficient recombinant protein production and secretion from nuclear transgenes in *Chlamydomonas reinhardtii*. J Biotechnol.

[36] Jaeger D, Baier T, Lauersen KJ (2019). Intronserter, an advanced online tool for design of intron containing transgenes. Algal Res.

[37] Kindle KL (1990). High-frequency nuclear transformation of *Chlamydomonas reinhardtii*. Proc Natl Acad Sci USA.

[38] Lauersen KJ, Kruse O, Mussgnug JH (2015). Targeted expression of nuclear transgenes in *Chlamydomonas reinhardtii* with a versatile, modular vector toolkit. Appl Genet Mol Biotechnol.

[39] Elbourne LDH, Tetu SG, Hassan KA, Paulsen IT (2017). TransportDB 2.0: a database for exploring membrane transporters in sequenced genomes from all domains of life. Nucleic Acids Res.

[40] Armenteros JJA, Sønderby CK, Sønderby SK, Nielsen H, Winther O (2017). DeepLoc: prediction of protein subcellular localization using deep learning. Bioinformatics.

[41] Krogh A, Larsson B, Von Heijne G, Sonnhammer ELL (2001). Predicting transmembrane protein topology with a hidden Markov model: application to complete genomes. J Mol Biol.

[42] Eddy SR (2011). Accelerated profile HMM searches. PLoS Comput Biol.

