# Supplementary material for: Farnesyl pyrophosphate compartmentalization in the green microalga Chlamydomonas reinhardtii during heterologous (E)-α-bisabolene production
Source: Microb Cell Fact. 2022 Sep 14;21:190. doi: 10.1186/s12934-022-01910-5 (PMC9472337; doi:10.1186/s12934-022-01910-5)
Supplement: Supplementary file 11 — Additional file 11: Figure S1. Attempts to increase flux towards cytosolic FPP by CrFPPS accumulation. Different orientations with the mCerulean3 reporter and co-expression of AgBS and corresponding bisabolene yields (fg cell−1) are depicted (constructs indicated on the left). Error bars represent 95% confidence intervals. Figure S2. Detection of full-length protein products of 3 independent cell lines transformed with various constructs. Each cell line is denoted by individual numbers, constructs are specified at the top, and corresponding bisabolene yields are depicted in Fig. 2. Total cellular proteins were run on a 10% SDS PAGE gel and subjected to Western blotting with α-GFP antibody. M marker (PageRuler prestained protein ladder, ThermoFisher Scientific). WT parental strain UVM4. P positive control expressing AgBS-mVenus, AgBS-mCerulean3, and AgBS-mRuby2 to the cytosol. Positive control and ERG20 protein products show previously observed pattern of protein breakage in the reporter [7]. Predicted protein sizes are: ~ 124.0 kDa for AgBS-mVenus, ~ 70.2 kDa for ERG20-mCerulean3, and ~ 67.7 kDa for mCerulean3-ERG20 which lacks a StrepII tag on its C terminus. Figure S3. Detection of full-length protein products of 3 independent cell lines transformed with various constructs. Each cell line is denoted by individual numbers, constructs are specified at the top, and corresponding bisabolene yields are depicted in Fig. 3. Total cellular proteins were run on a 10% SDS PAGE gel and subjected to Western blotting with α-GFP antibody. M marker (PageRuler prestained protein plus ladder, ThermoFisher Scientific). WT parental strain UVM4. Y mVenus expressed to the cytosol. Predicted protein sizes are: ~ 124.0 kDa for AgBS-mVenus, ~ 161.9 kDa for AgBS-mVenus-ERG20. Ponceau S stain is shown as loading control on the nitrocellulose membrane. Signal for construct vi, strain #3 was below detection limit, but bisabolene could be detected in GC–MS measurements, indicating full-length [file 12934_2022_1910_MOESM11_ESM.pdf]

**Supplemental Figures**

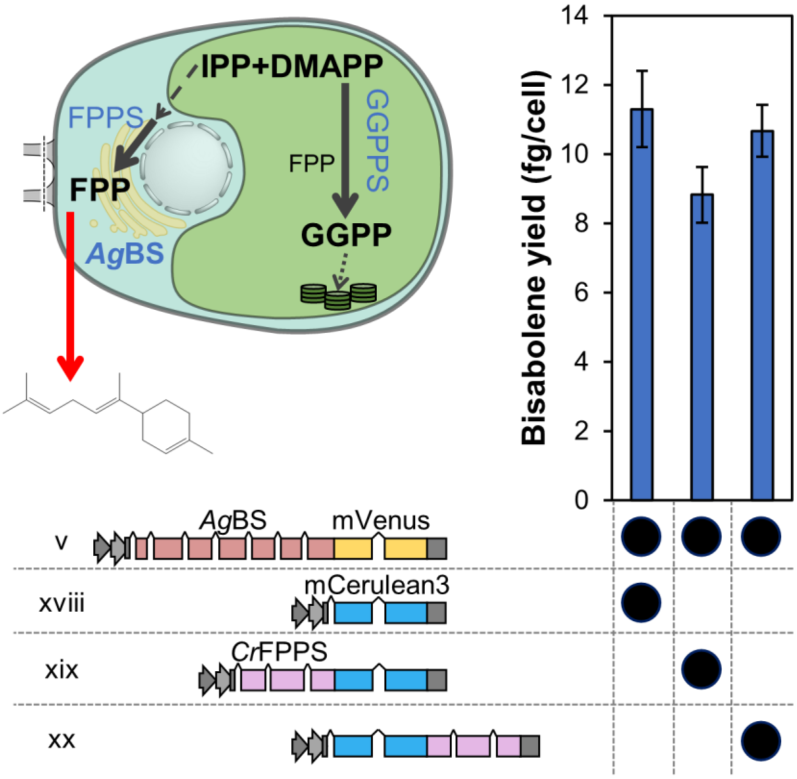


**Supplemental Figure 1.** Attempts to increase flux towards cytosolic FPP by *Cr*FPPS accumulation. Different orientations with the mCerulean3 reporter and co-expression of *Ag*BS and corresponding bisabolene yields (fg cell$^{-1}$) are depicted (constructs indicated on the left). Error bars represent 95 % confidence intervals.

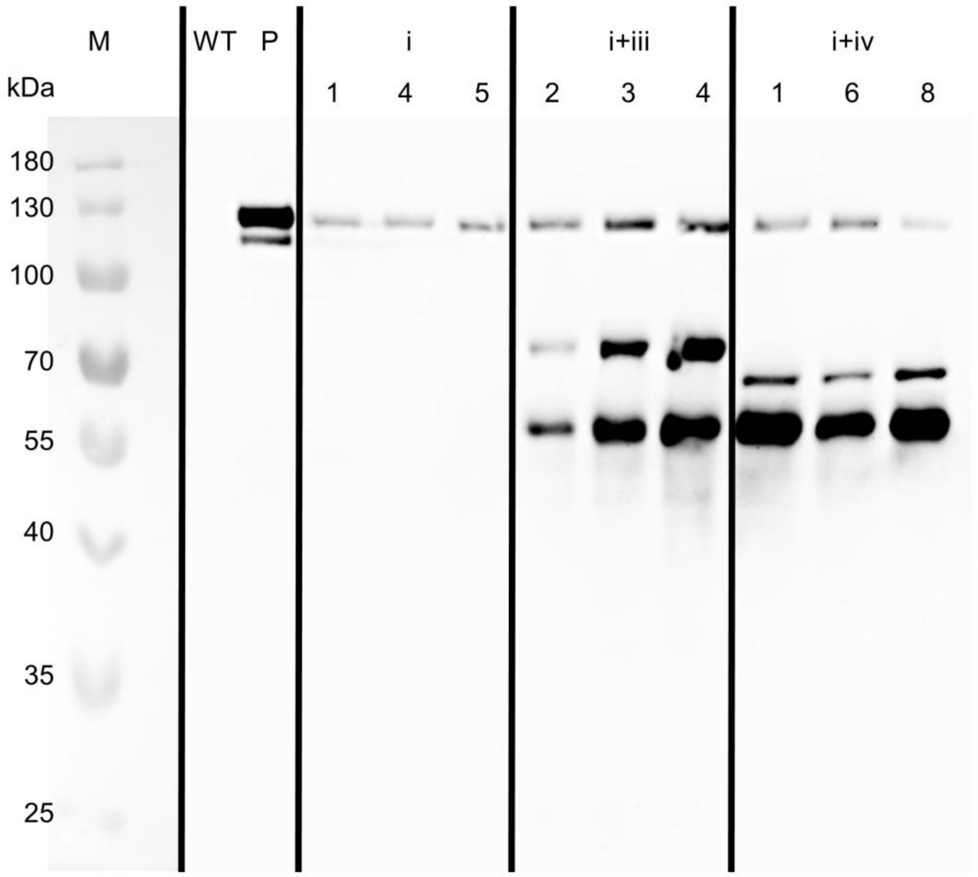


**Supplemental Figure 2.** Detection of full-length protein products of 3 independent cell lines transformed with various constructs. Each cell line is denoted by individual numbers, constructs are specified at the top, and corresponding bisabolene yields are depicted in Fig. 2. Total cellular proteins were run on a 10 % SDS PAGE gel and subjected to Western blotting with α-GFP antibody. **M** marker (PageRuler prestained protein ladder, ThermoFisher Scientific). **WT** parental strain UVM4. **P** positive control expressing *Ag*BS-mVenus, *Ag*BS-mCerulean3, and *Ag*BS-mRuby2 to the cytosol. Positive control and ERG20 protein products show previously observed pattern of protein breakage in the reporter [7]. Predicted protein sizes are: ~124.0 kDa for *Ag*BS-mVenus, ~70.2 kDa for ERG20-mCerulean3, and ~67.7 kDa for mCerulean3-ERG20 which lacks a StrepII tag on its C terminus.

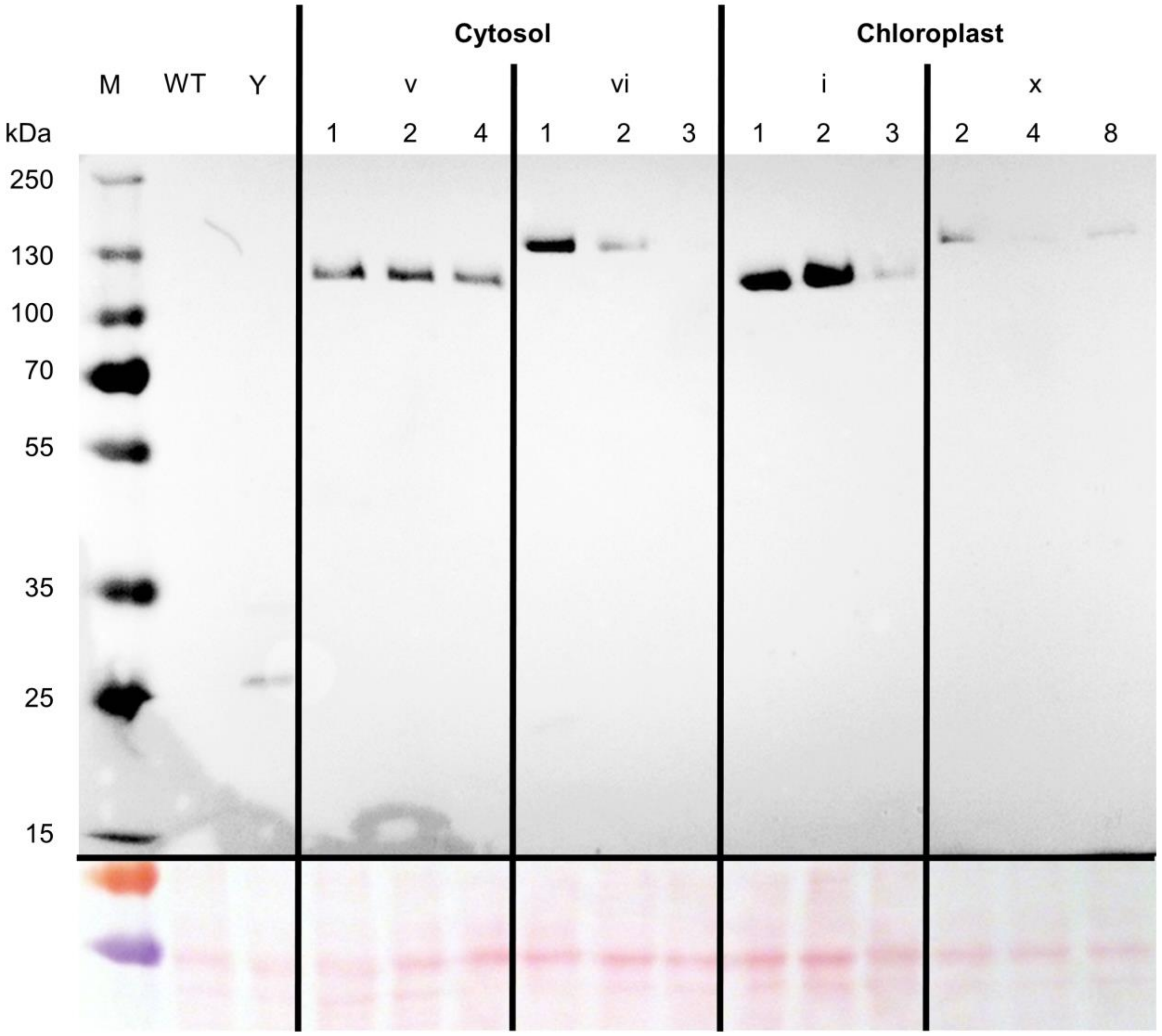


**Supplemental Figure 3.** Detection of full-length protein products of 3 independent cell lines transformed with various constructs. Each cell line is denoted by individual numbers, constructs are specified at the top, and corresponding bisabolene yields are depicted in Fig. 3. Total cellular proteins were run on a 10 % SDS PAGE gel and subjected to Western blotting with α-GFP antibody. **M** marker (PageRuler prestained protein plus ladder, ThermoFisher Scientific). **WT** parental strain UVM4. **Y** mVenus expressed to the cytosol. Predicted protein sizes are: ~124.0 kDa for *Ag*BS-mVenus, ~161.9 kDa for *Ag*BS-mVenus-ERG20. Ponceau S stain is shown as loading control on the nitrocellulose membrane. Signal for construct vi, strain #3 was below detection limit, but bisabolene could be detected in GC-MS measurements, indicating full-length expression.

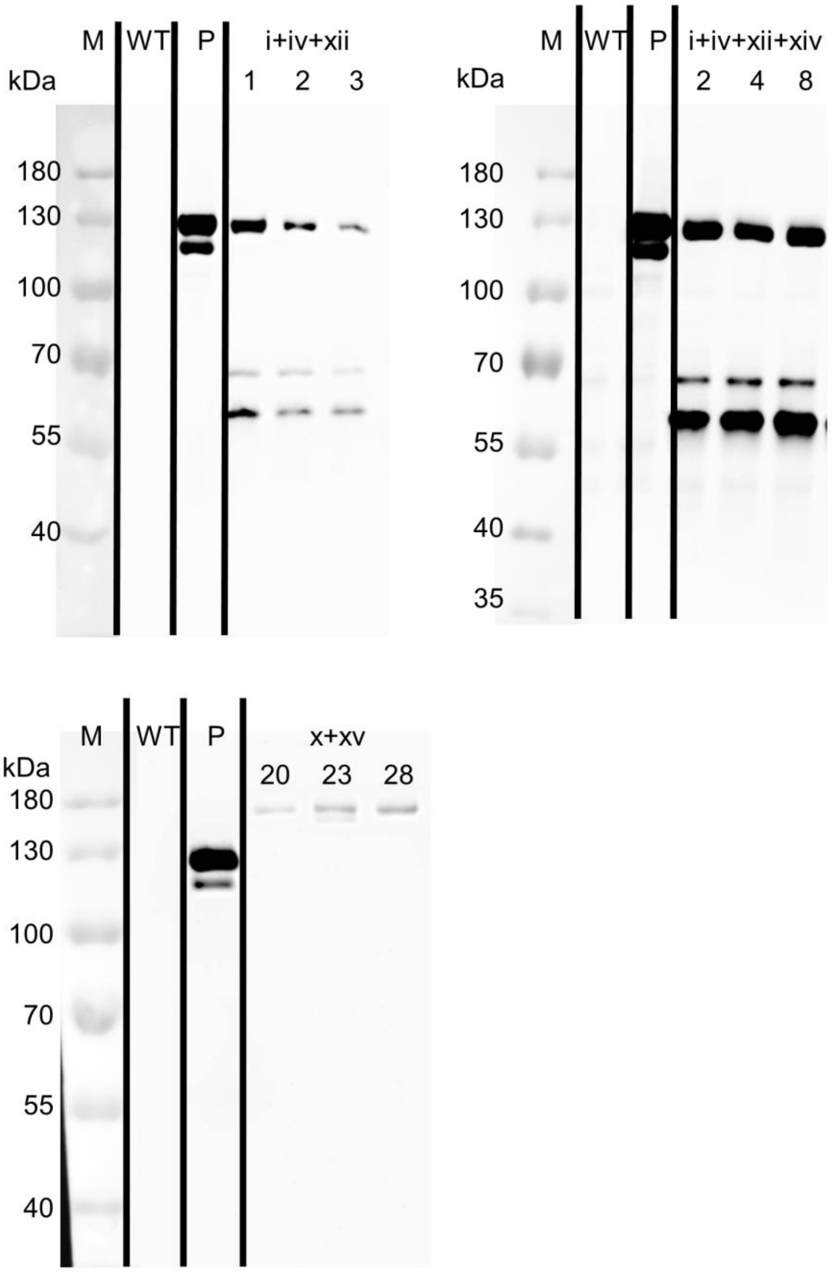


**Supplemental Figure 4.** Detection of full-length protein products of 3 independent cell lines transformed with various constructs. Each cell line is denoted by individual numbers, constructs are specified at the top, and corresponding bisabolene yields are depicted in Fig. 4. Total cellular proteins were run on a 10 % SDS PAGE gel and subjected to Western blotting with α-GFP and α-StrepII antibodies. **M** marker (PageRuler prestained protein ladder,

ThermoFisher Scientific). **WT** parental strain UVM4. **P** positive control expressing *Ag*BS-mVenus, AgBS-mCerulean3, and AgBS-mRuby2 to the cytosol. Positive control and ERG20 protein products show previously observed pattern of protein breakage in the reporter [7]. Predicted protein sizes are: ~124.0 kDa for *Ag*BS-mVenus, ~123.1 kDa for *Ag*BS-mCerulean3, ~120.9 kDa for *Ag*BS-mRuby2, ~123.7 kDa for *Ag*BS-aadA, ~67.7 kDa for mCerulean3-ERG20 which lacks a StrepII tag on its C terminus, ~161.9 kDa for *Ag*BS-mVenus-ERG20, and ~162.2 kDa for *Ag*BS-mCerulean3-ERG20.

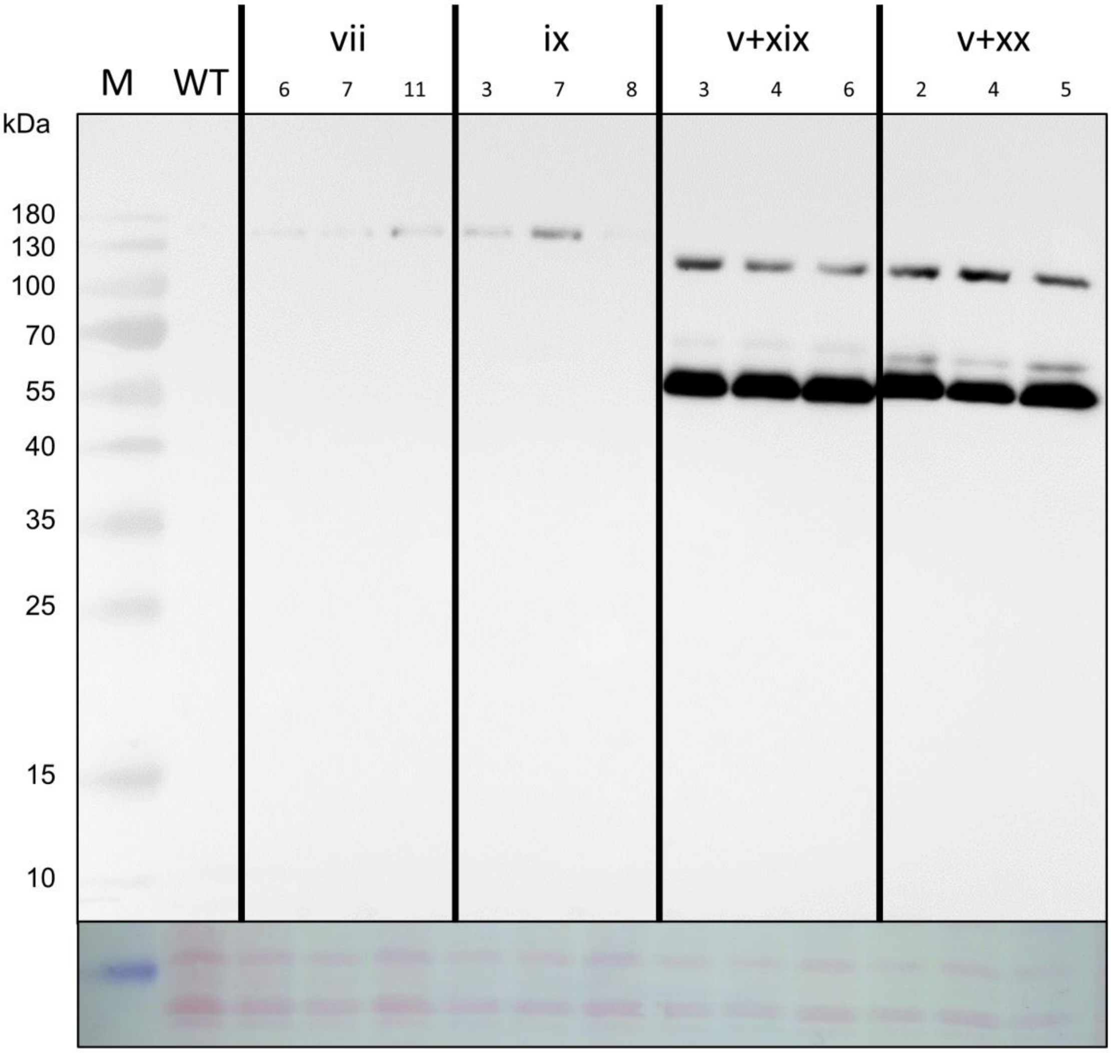


**Supplemental Figure 5.** Detection of full-length protein products of 3 independent cell lines transformed with various constructs. Each cell line is denoted by individual numbers, constructs are specified at the top, and corresponding bisabolene yields are depicted in Fig. 3 and Sup. Fig. 1. Total cellular proteins were run on a 10 % SDS PAGE gel and subjected to Western blotting with α-GFP antibodies. **M** marker (PageRuler prestained protein ladder, ThermoFisher Scientific). **WT** parental strain UVM4. *Cr*FPPS protein products show previously observed pattern of protein breakage in the reporter [7]. Predicted protein sizes are: ~160.9 kDa for *Ag*BS-mVenus-*Cr*FPPS, ~124.0 kDa for *Ag*BS-mVenus, ~68.5 kDa for *Cr*FPPS-mCerulean3, and ~67.0 kDa for mCerulean3-*Cr*FPPS which lacks a StrepII tag on its C terminus.

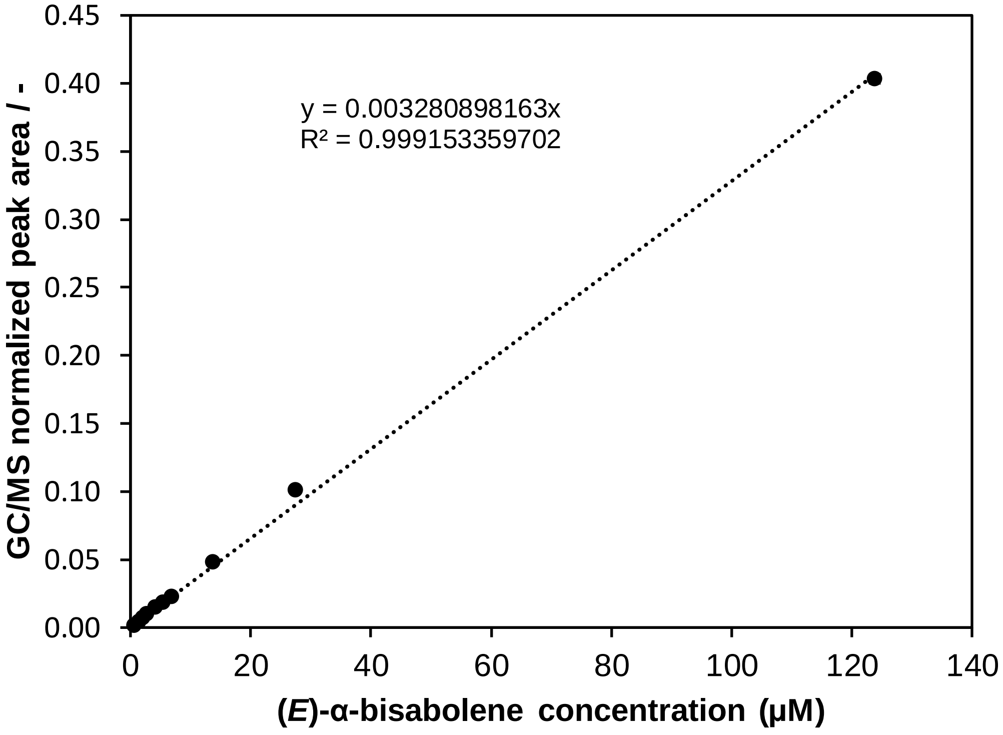


**Supplemental Figure 6.** Calibration line and correspondent equation for bisabolene quantification. GC/MS peak areas were normalized to the internal standard peak area, and plotted against the bisabolene concentration in dodecane.

## References

Lauersen, K. J., Baier, T., Wichmann, J., Wördenweber, R., Mussgnug, J. H., Hübner, W., Huser, T., & Kruse, O. (2016). Efficient phototrophic production of a high-value sesquiterpenoid from the eukaryotic microalga *Chlamydomonas reinhardtii*. *Metabolic Engineering*, *38*, 331–343. https://doi.org/10.1016/j.ymben.2016.07.013
